# Nodal Dependent Differential Localisation of Dishevelled-2 Demarcates Regions of Differing Cell Behaviour in the Visceral Endoderm

**DOI:** 10.1371/journal.pbio.1001019

**Published:** 2011-02-22

**Authors:** Georgios Trichas, Bradley Joyce, Lucy A. Crompton, Vivienne Wilkins, Melanie Clements, Masazumi Tada, Tristan A. Rodriguez, Shankar Srinivas

**Affiliations:** 1 Department of Physiology Anatomy & Genetics, University of Oxford, South Parks Road, Oxford, United Kingdom; 2 MRC Clinical Sciences Centre, Imperial College London, Hammersmith Hospital Campus, London, United Kingdom; 3 Department of Cell and Developmental Biology, University College London, London, United Kingdom; Duke University Medical Center, United States of America

## Abstract

The anterior visceral endoderm (AVE), a signalling centre within the simple epithelium of the visceral endoderm (VE), is required for anterior-posterior axis specification in the mouse embryo. AVE cells migrate directionally within the VE, thereby properly positioning the future anterior of the embryo and orientating the primary body axis. AVE cells consistently come to an abrupt stop at the border between the anterior epiblast and extra-embryonic ectoderm, which represents an end-point to their proximal migration. Little is known about the underlying basis for this barrier and how surrounding cells in the VE respond to or influence AVE migration. We use high-resolution 3D reconstructions of protein localisation patterns and time-lapse microscopy to show that AVE cells move by exchanging neighbours within an intact epithelium. Cell movement and mixing is restricted to the VE overlying the epiblast, characterised by the enrichment of Dishevelled-2 (Dvl2) to the lateral plasma membrane, a hallmark of Planar Cell Polarity (PCP) signalling. AVE cells halt upon reaching the adjoining region of VE overlying the extra-embryonic ectoderm, which displays reduced neighbour exchange and in which Dvl2 is excluded specifically from the plasma membrane. Though a single continuous sheet, these two regions of VE show distinct patterns of F-actin localisation, in cortical rings and an apical shroud, respectively. We genetically perturb PCP signalling and show that this disrupts the localisation pattern of Dvl2 and F-actin and the normal migration of AVE cells. In *Nodal* null embryos, membrane localisation of Dvl2 is reduced, while in mutants for the *Nodal* inhibitor *Lefty1*, Dvl2 is ectopically membrane localised, establishing a role for Nodal in modulating PCP signalling. These results show that the limits of AVE migration are determined by regional differences in cell behaviour and protein localisation within an otherwise apparently uniform VE. In addition to coordinating global cell movements across epithelia (such as during convergence extension), PCP signalling in interplay with TGFβ signalling can demarcate regions of differing behaviour within epithelia, thereby modulating the movement of cells within them.

## Author Summary

The orientation of the head-tail axis is determined during embryogenesis by the movements of a subset of cells called the AVE (anterior visceral endoderm). These cells migrate from their initial position within the simple epithelium of the visceral endoderm (VE) to a location from which they eventually induce anterior pattern in the adjacent epiblast. Little is understood about how AVE cells migrate within the VE, why they stop migrating where they do, and how surrounding cells in the VE respond to or influence AVE migration. In this study, we use time-lapse microscopy and high-resolution 3D reconstructions of protein localisation patterns to address these issues. Our results show that AVE cells move by exchanging neighbours within an intact epithelium. The limits of AVE migration are determined by regional differences in cell behaviour and protein localisation within an otherwise apparently uniform VE. Finally, we examine the role of planar cell polarity (PCP) signalling, which is responsible for coordinating morphogenetic events across different epithelia. We show that in addition to this traditional role in coordinating global cell movements, PCP signalling along with TGFβ signalling can demarcate regions of differing behaviour within epithelia, thereby modulating the movement of cells within them.

## Introduction

The anterior visceral endoderm (AVE) is a specialised sub-set of the visceral endoderm (VE) that is responsible for inducing anterior pattern in the underlying epiblast (reviewed in [1]–[4]). It is induced at the distal tip of the egg-cylinder in a Nodal dependent manner [5]–[7]. From this initial distal position, it migrates directionally to the future embryonic anterior, thereby properly orientating the anterior-posterior axis of the embryo. Once positioned over the future anterior epiblast, the AVE represses posterior markers in the abutting epiblast, thereby restricting their expression to the opposite side of the epiblast cup, where the primitive streak later forms [8].

*Dkk1*, an inhibitor of canonical Wnt signalling, has been demonstrated to act as a guidance cue for AVE migration [9]. An asymmetry in the early distal VE expression of the *Nodal* antagonists *Lefty1* and *Cer1* has been reported to result in differential proliferation in the VE, leading to the initial displacement of the AVE towards the future anterior [10]. There are no reports of pre-gastrulation developmental abnormalities in either *Lefty1* or *Cer1* null mutant embryos [11]–[15]. However, *Lefty1;Cer1* double mutants show an abnormal accumulation of cells in the anterior region of the VE as early as 6.5 days *post coitum* (dpc) (just prior to gastrulation) as well as an expansion and occasional duplication of the primitive streak at gastrulation stages [16].

Planar Cell Polarity (PCP) signalling is responsible for coordinating morphogenetic events across fields of cells, such as the regular orientation of bristles on the fly wing, or polarised mediolateral intercalation during embryonic axis elongation by convergent extension [17]–[20]. Dishevelled (Dvl) is a key mediator of Wnt signalling through both canonical and PCP pathways. Dvl translocation to the cell membrane is a hallmark of PCP signalling [21],[22]. Another core PCP molecule is flamingo, an atypical member of the E-Cadherin super-family. Flamingo is a 7-pass trans-membrane molecule that is essential for normal PCP function, though the exact mechanism by which it acts remains unclear [23]. One of the primary modes of action of PCP signalling is through non-muscle myosin IIA and F-actin, that together facilitate junctional remodelling in epithelia [24]–[26]. Mutants of *Nap1*, a regulator of Actin branching, have AVE migration defects [27]. The small GTPase Rac1 modulates cytoskeletal dynamics in response to PCP signals. *Rac1* mutants have also recently been shown to have AVE migration defects [28].

Time-lapse studies show that the movement of AVE cells to the future anterior is an active process that is completed in the order of 4 to 5 h and that AVE cells come to an abrupt halt at the boundary between the epiblast and the extraembryonic ectoderm (ExE) [29]. The VE remains a monolayer during AVE migration, suggesting that AVE cells migrate through the surrounding VE cells rather than on top of them [29]. However, since it is only AVE cells that have been visualised to date, very little is known about how surrounding VE cells respond to or possibly influence AVE migration. For example, it is unknown if the cells surrounding AVE cells are also motile and whether VE cells “ahead” of the migrating AVE are displaced onto the ExE, displaced laterally, or removed in some other way such as apoptosis. Why AVE cells stop moving proximally upon reaching the ExE is also unknown, particularly given that the VE overlying the epiblast and ExE are part of a single continuous sheet.

Using time-lapse microscopy to record the behaviour of VE cells, we show that those cells overlying the epiblast exchange neighbours through cell intercalation, while cells in the VE overlying the ExE are relatively static in their behaviour. This difference in behaviour correlates with regional differences in the localisation of F-actin and non-muscle myosin IIA. Dishevelled-2 (Dvl2) is membrane localised specifically in the VE overlying the epiblast, suggestive of active PCP signalling in this region. Genetically perturbing Dvl2 localisation leads to the abnormal migration of AVE cells onto the ExE. Membrane localisation of Dvl2 is reduced in *Nodal* mutants and ectopically increased in mutants of the *Nodal* inhibitor *Lefty1*, suggesting a way by which these key patterning molecules might also influence morphogenetic events.

## Results

### The VE Retains Epithelial Integrity During AVE Migration

Our previous time-lapse studies of AVE cells show them migrating in a manner reminiscent of fibroblasts, with cellular projections predominantly in the direction of migration [29]. To determine if AVE cells might be undergoing an epithelial to mesenchymal transition during migration and to determine if the end-point to their migration might be a manifestation of differences in the structure of the VE epithelium, we visualised the epithelial apical junctional markers ZO-1 (tight junctions) and E-cadherin (adherens junctions) at different stages of AVE migration. To visualise the AVE, we used embryos carrying a *Hex-GFP* reporter transgene that labels AVE cells [30]. To obtain information about the three-dimensional pattern of distribution of these molecules in the context of the whole embryo, we captured image volumes of entire embryos by confocal microscopy and visualised the data as opacity rendered 3D representations (Movie S1).

**Figure 1:**
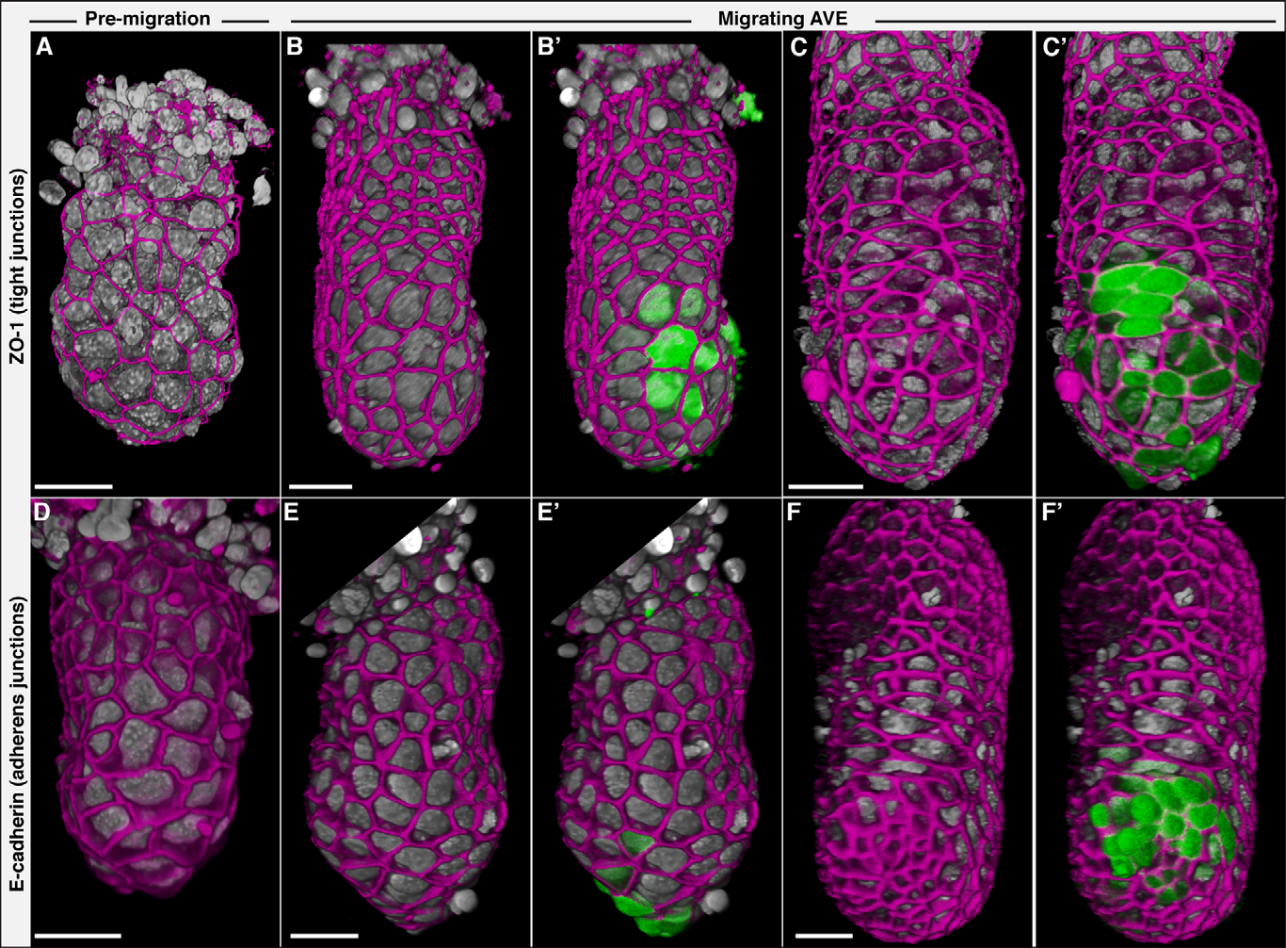
Localisation of ZO-1 and E-cadherin at different stages of AVE migration. Volume rendering of the tight junction marker ZO-1 (A–C', magenta) and E-cadherin (D–F', magenta) in representative wild-type embryos. Nuclei are in grey. (B', C', E', F') show the respective embryos with Hex-GFP marking AVE cells (green), to indicate the stage of AVE migration. Prior to DVE induction (A, D), during AVE migration (B, E) and when the AVE has reached the anterior extent of its migration (C, F), these two epithelial junctional markers are expressed in an uninterrupted manner throughout the VE, even amongst migrating AVE cells. All embryos are anterior views except for those in (A) and (D), where it has not been defined yet. Scale bars  = 50 µm.

Both ZO-1 and E-cadherin were detected continuously and uniformly along cell-cell junctions throughout the VE, at all stages of AVE migration (Figure 1 and Movie S2). We did not detect any discontinuity in ZO-1 or E-cadherin even amongst migrating AVE cells (Figure 1B–F'), indicating that the AVE and surrounding VE retain epithelial integrity during AVE migration. These results are also consistent with similar findings recently published by Migeotte et al. [28].

### Intercalation and Dynamic Behaviour of Cells in the VE

To visualise surrounding VE cells during AVE migration, we used time-lapse microscopy and DIC optics to monitor apical cell borders of the surface VE. We captured images from five focal planes at each time-point so cell outlines could be visualised unambiguously, and with a 15 min interval to achieve sufficient temporal resolution to follow individual cells from one time-point to the next. We used *Hex-GFP* transgenic embryos, so that AVE migration could be monitored. Four embryos remained in the field of view and in focus from the start of AVE migration to the finish and were used to quantify VE cell movement characteristics. Due to the strong curvature of the surface of the egg-cylinder, cell outlines in the “peripheral” regions of the VE could not be easily discerned and therefore cells in this region were not included in analyses.

Cells of the VE overlying the epiblast (referred to hereafter as Epi-VE) showed dramatically different behaviour as compared to cells of the VE overlying the ExE (referred to hereafter as ExE-VE).

**Figure 2:**
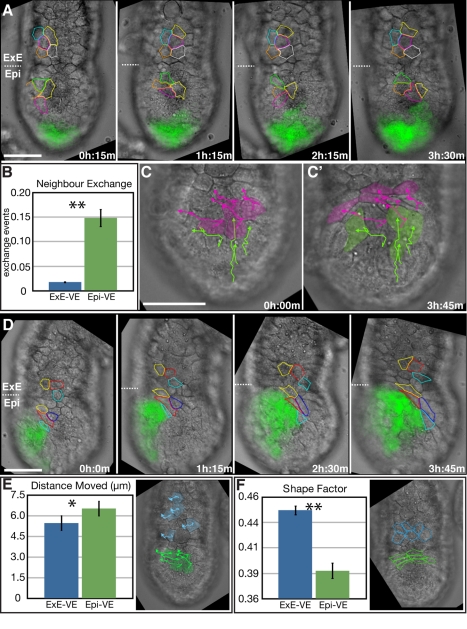
Cell intercalation and shape changes during AVE migration. (A) Specific frames from a time-lapse sequence of surface VE during migration of AVE cells (green). Images were captured at 5 focal planes every 15 min. Representative cells are outlined in different colours so they can be easily followed. Dotted lines indicate the border between the Epi-VE and ExE-VE. AVE migration is accompanied by a great deal of cell intercalation among Epi-VE cells, resulting in neighbour exchange. In contrast, cells of the ExE-VE are relatively static. (B) Average number of neighbour exchange events in the Epi-VE versus ExE-VE (*n* = 46 and 44 cells, respectively, from 4 embryos). Exchange events were normalised for the number of cells tracked and expressed as the number of exchange events per cell per time interval (15 min for the time-lapse data). There were on average 0.15 exchange events per cell (i.e., one exchange event involving two cells, among every ∼7 cells) in the Epi-VE in every 15 min period. (C, C') First and last frames of a time-lapse sequence showing the tracks of representative cells in the Epi-VE. *Hex-GFP* expressing AVE cells are marked green and non-Hex-GFP Epi-VE cells are magenta. (D) Another embryo, illustrating dramatic changes in cell shapes in the Epi-VE during AVE migration. Non-Hex-GFP positive cells of the Epi-VE (outlined) are unable to move onto the ExE-VE. (E) Comparison of the average distance moved by cells in one time interval (15 min) in the Epi-VE versus ExE-VE (*n* = 46 and 44 cells, respectively, from four embryos), with an illustrative example of tracks of cells in the two regions. (F) Comparison of average shape factor of cells in the Epi-VE versus ExE-VE (*n* = 46 and 44 cells, respectively, from four embryos), with illustrative example of cells shapes in the two regions. Shape factor can range form 0 to 1, higher values being closer to circles or regular shapes than lower values. * indicates *p*<0.005 and ** indicates *p*<0.0001 (student's *t* test). Scale bar  = 50 µm.

We observed extensive neighbour exchange as a result of cell intercalation among the *Hex-GFP* negative cells of the Epi-VE just “ahead” of the migrating AVE (Figure 2A, and Movie S3). We also occasionally observed neighbour exchange between AVE cells and surrounding *Hex-GFP* negative Epi-VE cells. On average, 2 out of every 7 Epi-VE cells were involved in a neighbour exchange event in a 15 min time period (Figure 2B). In contrast, cells of the ExE showed almost no neighbour exchange (*n* = 46 and 44 cells for the Epi-VE and ExE-VE, respectively, from 4 embryos; *p*<0.0001, Student's *t* test; Figure 2B). Cells of the Epi-VE and ExE-VE did not intermingle, creating an interface of differing cell behaviour between these two regions.

Cell tracking showed that *Hex-GFP* negative Epi-VE cells ahead of the AVE moved in a directional manner similar to AVE cells (Figure 2C and C' and Movie S4). Rather than simply moving aside to make way for migrating AVE cells, these cells remain ahead of AVE cells by moving proximally, until they reached the border with the ExE-VE. Once there, they become severely distorted in shape and start to move laterally, being unable to move onto the ExE (Figure 2D and C', and Movie S5). This is similar to the behaviour AVE cells show upon reaching the ExE, where the leading AVE cells change shape and start to move laterally [29].

Compared with Epi-VE cells, ExE-VE cells showed significantly less average movement (*n* = 46 and 44 cells for the Epi-VE and ExE-VE, respectively, from 4 embryos; *p*<0.005, Student's *t* test; Figure 2E). Cells of the Epi-VE were irregular in shape and appeared very labile, changing shape continuously. In comparison, cells of the ExE-VE were more regular in outline and did not change shape, remaining relatively static. To quantify this difference we determined the “shape factor” of cells, which is a measure of how close to being a perfect circle a shape is. Values can range from 0 to 1, with higher values indicative of a more circular or regular shape. Consistent with ExE-VE cells being more regular in outline, the average shape factor of ExE-VE cells was significantly higher than that of Epi-VE cells (*n* = 46 and 44 cells for the Epi-VE and ExE-VE, respectively, from 4 embryos; *p*<0.0001, Student's *t* test, Figure 2F and Movies S3, S4, and S5).

### F-Actin and Myosin IIA Show Regional Differences in Localisation

Non-muscle myosin IIA and F-actin are molecular motors that facilitate cell shape changes and intercalation in epithelia [25],[26]. To determine if they might play a role in the cell intercalation we observe in the Epi-VE, we stained *Hex-GFP* embryos at various stages of AVE migration with Phalloidin (which stains F-actin) and an antibody against myosin IIA.

**Figure 3:**
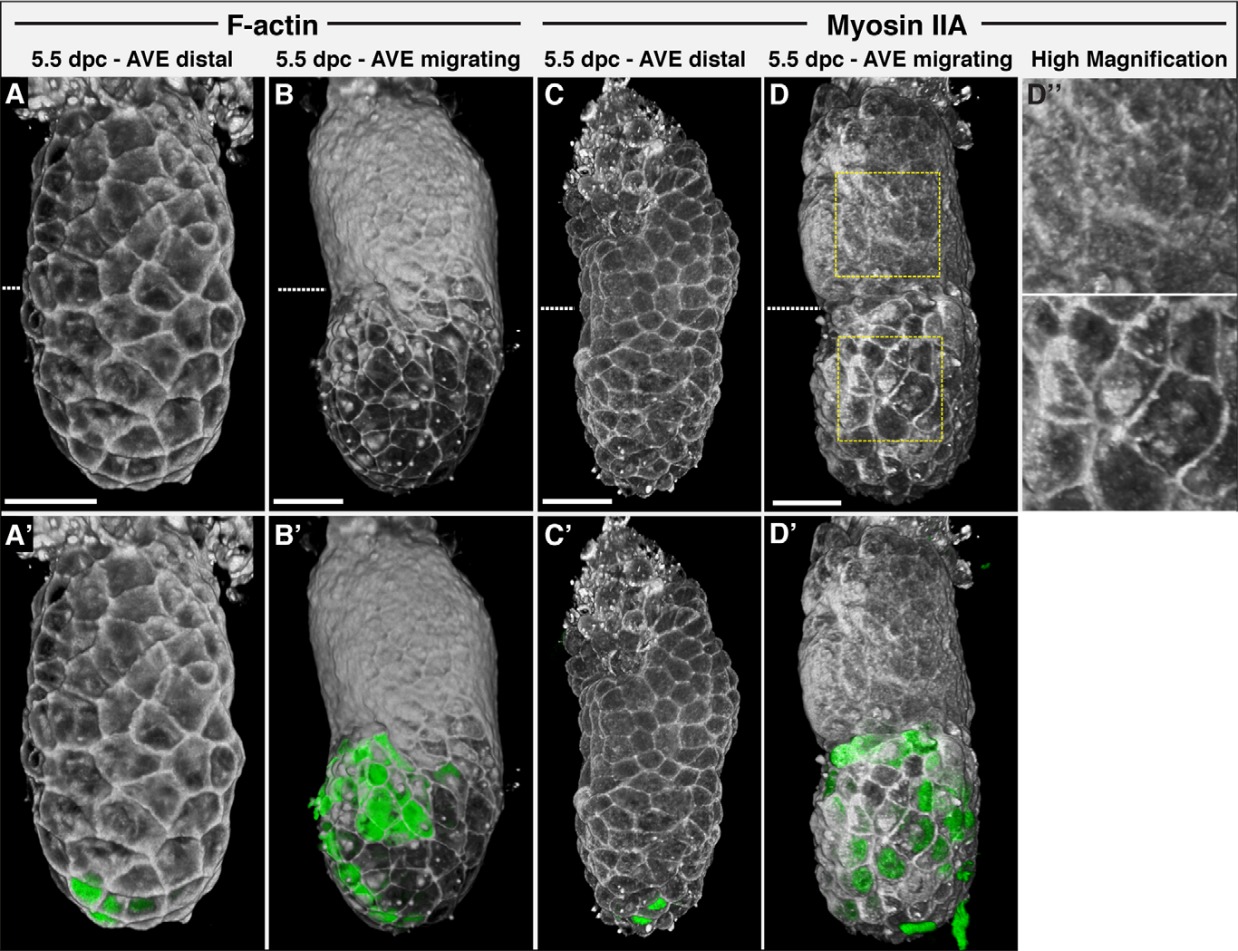
F-actin and myosin IIA localisation at different stages of AVE migration. (A–B) Volume rendering of F-actin localisation in representative wild-type embryos at early and late 5.5 dpc, respectively. F-actin is initially localised to cortical rings in cells throughout the VE but by the time the AVE has migrated to the anterior comes to be localised to an apical shroud specifically in the ExE-VE, while remaining in cortical rings in the Epi-VE. (C) Prior to AVE migration, myosin IIA is localised to cortical rings throughout the VE. (D) During AVE migration, myosin IIA remains in distinct cortical rings throughout the Epi-VE but is much less distinctly membrane localised in the ExE-VE. (D″) High magnification views of the boxed regions in (D). (A', B', C', D') show the respective embryos with Hex-GFP marking AVE cells (green), to indicate the stage of AVE migration. Anterior is to the left. Dotted lines indicate the border between the Epi-VE and ExE-VE. Scale bars  = 50 µm.

**Figure 4:**
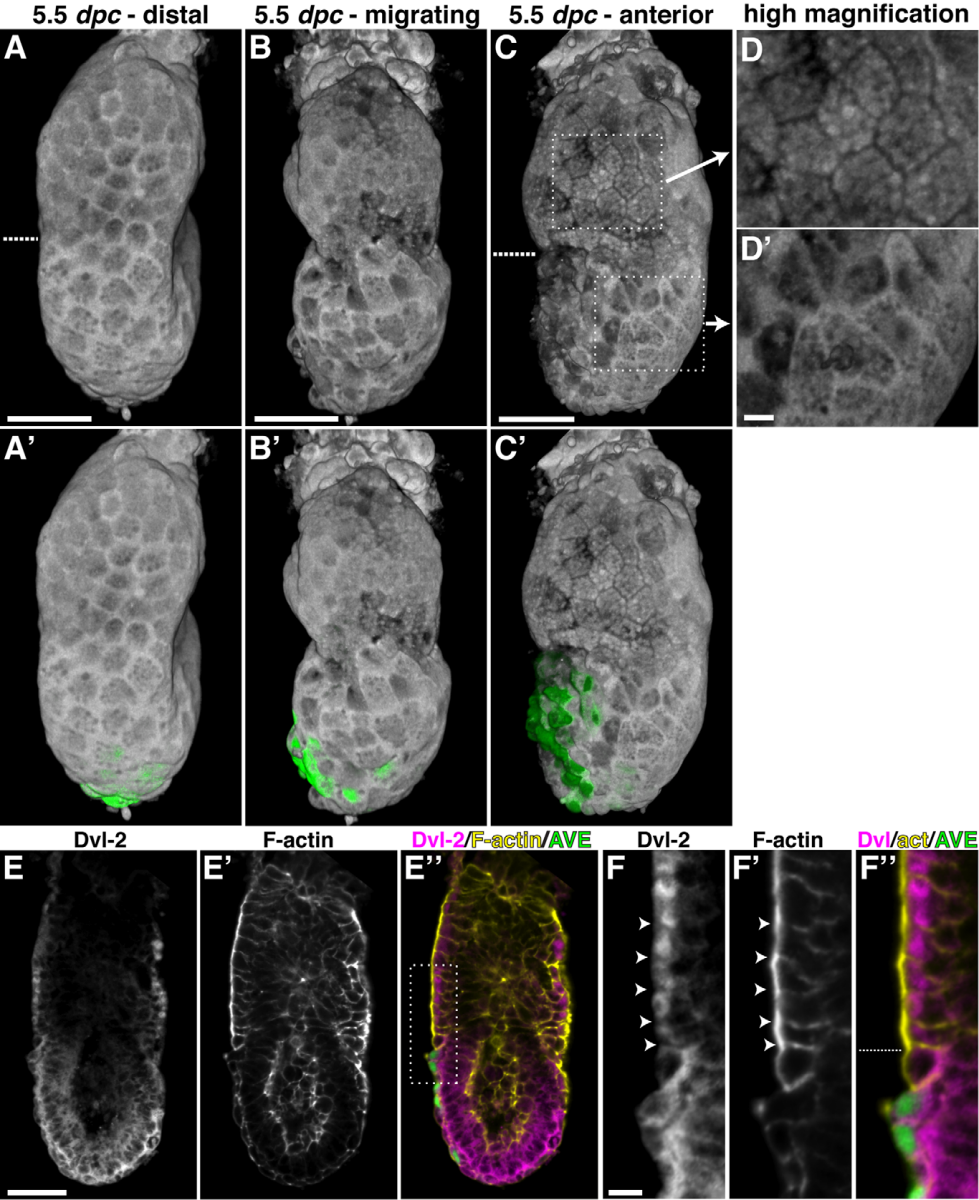
Dvl2 localisation at different stages of AVE migration. (A, B, C) Volume rendering of endogenous Dvl2 protein in representative 5.5 dpc wild-type embryos prior to AVE migration, during migration and after the AVE has reached the endpoint of migration. At early stages, Dvl2 is membrane localised throughout the VE, but present at slightly lower levels in the ExE-VE. During the course of AVE migration and after, Dvl2 is progressively down-regulated in the ExE-VE, and specifically excluded from the plasma membrane. (A', B', C') show the respective embryos with Hex-GFP marking AVE cells (green), to indicate the stage of AVE migration. (D, D') High magnification views of the boxed regions in (C), showing membrane exclusion and enrichment of Dvl2 in the ExE-VE and Epi-VE, respectively. (E, E', E″) Optical section of a late 5.5 dpc wild-type embryo showing Dvl2, F-actin, and a merge of Dvl2 in magenta, F-actin in yellow, and AVE cells expressing Hex-GFP in green. Regions with relatively low Dvl2 levels show elevated levels of F-actin overall, with the F-actin being more distinctly apically localised in the ExE-VE. (F, F', F″) High magnification images of the region outlined by the dotted lines in E″. Dvl2 is specifically diminished in the lateral membrane in cells of the ExE-VE (arrowheads in F, with corresponding regions of actin localisation marked by arrowheads in F'). Dotted lines indicate the border between the Epi-VE and ExE-VE. The scale bars represent 50 µm in (A–C, E) and 10 µm in (D, F).

Opacity rendering showed that in early 5.5 dpc embryos (prior to AVE migration), F-actin was expressed at uniform levels throughout the VE, localised to cortical rings around cells (Figure 3A, A'). During AVE migration, however, a difference in F-actin levels and localisation in the Epi-VE and ExE-VE became evident. F-actin was present in cortical rings in both regions but, in addition, was greatly enriched in the apical cell cortex specifically in the ExE-VE, forming a shroud across this region in opacity renderings (Figure 3B, B' and Movie S6). This actin shroud was found to be in place well before AVE cells reached the end-point of migration. In contrast, in the behaviourally labile cells of the Epi-VE, F-actin was lower in general, remained depleted in the apical cortex, and was specifically restricted to the lateral cell cortex. This broad difference in F-actin localisation was also observed in 6.25 dpc and 7.5 dpc embryos (Figure S1A and D). Optical sections showed that F-actin was expressed at higher levels in the ExE-VE than Epi-VE and verified its apical localisation in the ExE-VE (Figure 4E', F'). F-actin was detected in both the Epiblast and ExE. In the Epiblast, it was enriched particularly in the apical domain of epiblast cells, facing the pro-amniotic cavity (Figure 4E').

Myosin IIA showed a localisation pattern similar to F-actin. Prior to AVE migration, it was present in cortical rings throughout the entire VE (Figure 3C, C„). During migration, it remained localised to cortical rings in the Epi-VE. However, in the ExE-VE, it was present in an apical shroud, though not quite as distinctly as that observed with F-actin (Figure 3D–D„).

We did not detect any difference in localisation of F-actin or Myosin IIA between AVE and surrounding Epi-VE cells.

### Dvl2 Shows Regional Differences in Localisation

PCP signalling is responsible for coordinating morphogenetic events across epithelia and can act by modulating F-actin and non-muscle myosin localisation and function [24]. Dvl translocation to the cell membrane is a hallmark of PCP signalling [21],[22],[31]. To determine if PCP signalling might be involved in AVE migration, we assayed the sub-cellular localisation of Dvl2 by immunostaining *Hex-GFP* embryos at different stages of AVE migration with an antibody against Dvl2.

Opacity rendered volumes showed that in early 5.5 dpc embryos (in which the AVE was still at the distal tip, prior to migration), Dvl2 was detected in the lateral cell membrane in both the Epi-VE and ExE-VE, though it was present at slightly lower levels in the ExE-VE, particularly in the region adjacent to the Epi-VE (Figure 4A, A'). In embryos in which the AVE is in the process of migrating (as shown by Hex-GFP positive AVE cells), Dvl2 was differentially localised in the two regions of the VE. In the Epi-VE, Dvl2 remained enriched in lateral cell membranes. In the ExE-VE, however, Dvl2 was greatly reduced in the lateral membrane. In some ExE-VE cells, particularly those close to the border with the Epi-VE, Dvl2 was reduced throughout the cell, in both the membrane and cytoplasm (Figure 4B, B'). This difference in localisation increased progressively during AVE migration such that in embryos in which the AVE had reached the proximal extent of migration, in the Epi-VE Dvl2 remained elevated in the cell membrane, while in the ExE-VE it was excluded from the cell membrane (Figure 4C–D') or downregulated throughout the cell (unpublished data). By 6.25 dpc, Dvl2 was almost completely absent from the ExE-VE, though it remained membrane enriched in the Epi-VE (Figure S1B). This region specific difference in Dvl2 localisation was also observed in 7.5 dpc embryos (Figure S1E). We did not detect any difference in localisation of Dvl2 between AVE and surrounding Epi-VE cells.

Optical sections revealed that Dvl2 was expressed in the epiblast but showed much lower expression in the ExE (Figure 4E).

Consistent with a role for Dvl2 in F-actin remodelling, we found that the levels and localisation differences of the two are complementary. In those regions where Dvl2 was downregulated or excluded from the plasma membrane (ExE-VE), F-actin was upregulated and more apically localised. Conversely, where Dvl2 was enriched in the lateral plasma membrane (Epi-VE), F-actin was localised to cortical rings (Figure 4E–F„ and Figure S1C and F).

### Disruption of PCP Signalling Perturbs Dvl2 Localisation and AVE Migration

To directly test the involvement of PCP signalling in AVE migration, we engineered a mouse line in which PCP signalling is perturbed. Drosophila *Flamingo* is essential for establishing PCP [23]. The mouse homologue *Celsr1* is expressed at 5.5 dpc [32] and at later stages is essential for PCP dependent processes like the polarization of stereocilia in the inner ear [33]. Expression of a membrane tethered C-terminal fragment of the zebrafish Celsr1 protein disrupts PCP dependent convergent extension and Frizzled dependent membrane localisation of Dishevelled in zebrafish embryos by interfering with endogenous Celsr1 function [34].

We therefore engineered a near-identical construct, but using the mouse Celsr1 sequence (Figure S2A). When injected into zebrafish embryos, this construct recapitulated the convergent extension defects caused by the zebrafish version (Figure S2B). We next engineered mice in which this fusion construct was knocked into the ubiquitously expressed *ROSA26* locus (Figure S2C–E).

**Figure 5:**
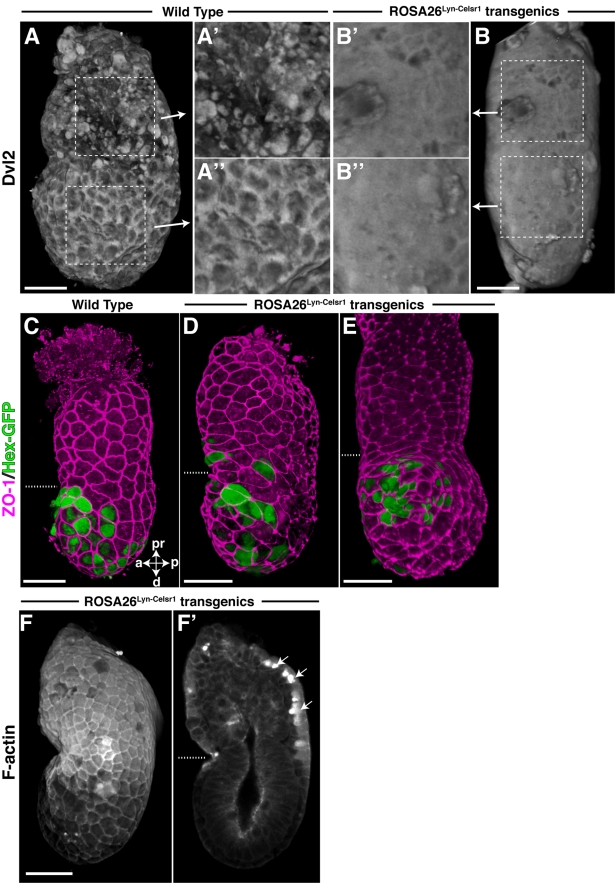
PCP defects in *ROSA26^Lyn-Celsr1^* embryos. (A, B) Volume rendering of Dvl2 localisation in a 5.75 dpc *ROSA26^Lyn-Celsr1^* embryo and wild-type litter-mate. (A', A″) High magnification images of the boxed regions in (A). (B', B″) High magnification images of the boxed regions in (B). Dvl2 is less distinctly membrane localised than normal in the Epi-VE (compare A″ with B″) and abnormally persistent in the ExE-VE (compare A' with B'). (C) Lateral view of volume rendering of a representative wild-type embryo illustrating normal migration of the AVE, as a contained group of cells that do not move into the ExE-VE. (D) *ROSA26^Lyn-Celsr1^* embryo showing abnormal AVE migration into the ExE-VE. (E) *ROSA26^Lyn-Celsr1^* embryo showing an abnormal swirling pattern of AVE cells, reminiscent of whorls of bristles and hair seen in PCP mutant flies and mice. Cell outlines in (C–E) are visualised by ZO-1 stain (magenta), while AVE cells are visualised by the expression of Hex-GFP (green). Dotted lines indicate the border between the Epi-VE and ExE-VE. (F, F') Opacity rendering of F-actin localisation in a *ROSA26^Lyn-Celsr1^* embryo showing attenuation of the apical F-actin shroud in the ExE-VE. (F') Optical section of the *ROSA26^Lyn-Celsr1^* embryo showing abnormal F-actin localisation. The arrows point to anomalous cytoplasmic F-actin aggregates. Scale bars  = 50 µm. pr, proximal; d, distal; a, anterior; p, posterior.

Mice heterozygous and homozygous for the *ROSA26^Lyn-Celsr1^* modification were born in Mendelian frequencies (unpublished data). To determine if PCP signalling was disrupted in transgenic embryos, we assayed the sub-cellular localisation of Dvl2 and F-actin in 5.75 dpc embryos. All *ROSA26^Lyn-Celsr1/Lyn-Celsr1^* embryos (*n* = 4) and half of the *ROSA26^Lyn-Celsr1/wt^* embryos (*n* = 8) showed abnormal localisation of Dvl2 and F-actin in opacity renderings. The normal membrane enrichment of Dvl2 was repressed in cells of the Epi-VE, with it instead being found in an abnormally diffuse pattern throughout the VE. In addition, in the ExE-VE there was an abnormal persistence of cytoplasmic Dvl2 at later stages, when it should have been downregulated (compare Figure 5A and B). Dvl2 was present in the epiblast as normal but was abnormally expressed in the ExE as well. We observed a similar disruption in the localisation of another core PCP member, Vangl2. In wild-type embryos Vangl2 was membrane enriched in the Epi-VE while in *ROSA26^Lyn-Celsr1^* transgenics, the membrane enrichment of Vangl2 in the Epi-VE was significantly repressed (Figure S3).

The apical shroud of F-actin in the ExE-VE was attenuated (Figure 5F). In addition, we found greater levels of cytoplasmic actin stain and abnormal cytoplasmic aggregates of F-actin (arrows in Figure 5F' and Movie S7), which are never seen in wild-type embryos. The enrichment of F-actin to the apical domain of epiblast cells was also markedly reduced (Figure 5F').

The repression of membrane localisation of Dvl2 and Vangl2 in *ROSA26^Lyn-Celsr1^* embryos indicates that PCP signalling has been disrupted. To determine if this is accompanied by any AVE migration defects, we crossed the *ROSA26^Lyn-Celsr1^* line to the *Hex-GFP* line to visualise the AVE. We examined opacity renderings of 5.75 dpc embryos stained for ZO-1 to visualise cell borders in the VE. Six out of 11 *ROSA26^Lyn-Celsr1/Lyn-Celsr1^* embryos (55%) and six out of 14 *ROSA26^Lyn-Celsr1/wt^* embryos (43%) showed AVE migration defects (*p* = 0.01 and 0.03, respectively, chi-square test, compared to 8 wild-type littermates). In contrast to wild-type embryos in which the AVE forms a contained patch of cells at the end of migration, none of which move onto the ExE (Figure 5C), in *ROSA26^Lyn-Celsr1^* transgenic embryos, AVE cells were found to spill over onto the ExE or spread much more broadly in the VE than normal (six of the 12 transgenic embryos with a migration defect) (Figure 5D and Movies S8 and S9). Nine of the 12 embryos showed abnormal swirling arrangements of AVE cells (Figure 5E and Movie S10), reminiscent of the disordered whorls of wing bristles and hair, respectively, in PCP mutants in Drosophila [35] and mice [36]. These two phenotypes were found to overlap in three embryos. The AVE “overmigration” phenotype in *ROSA26^Lyn-Celsr1^* mutant embryos was also detected in stains for the AVE marker *Cer1* (Figure S2F). These defects in AVE migration did not result in abnormal expression of the posterior epiblast marker *eomes* (Figure S2G) [37].

### Dvl2 Localisation Is Altered in Nodal and Lefty1 Mutants

The TGFβ molecule Activin causes membrane localisation of *Xenopus* dishevelled in *X. laevis* animal cap cells [38]. Mouse Nodal is closely related to Activin and plays a central role in AVE induction and migration [5],[39]. To determine if Nodal might be involved in regulating the differential localisation of Dvl2 that we observe, we looked at Dvl2 localisation in *Nodal* null (*Nodal^lacZ/lacZ^*) embryos [40] at 5.75 dpc and 6.25 dpc.

**Figure 6:**
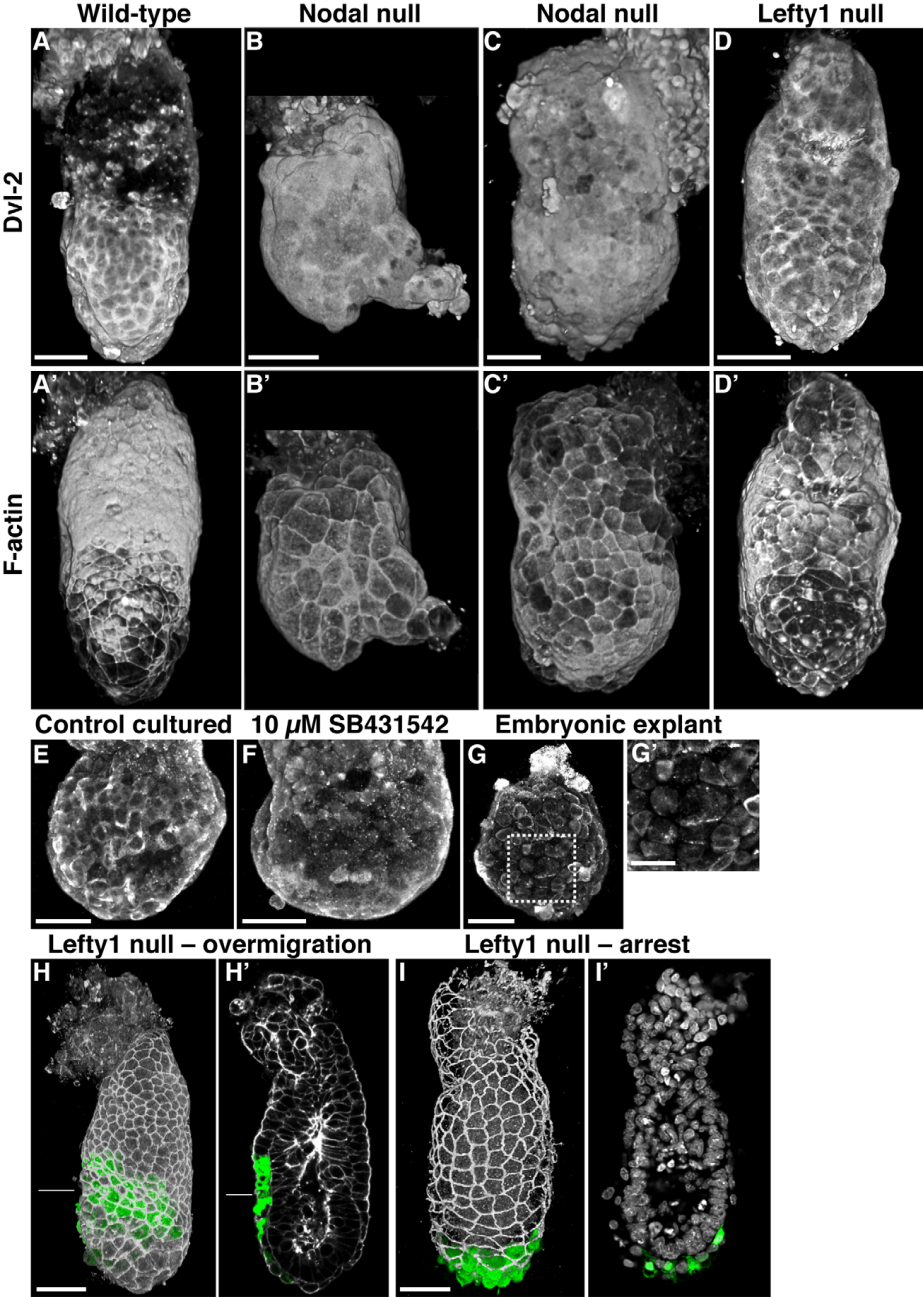
Defects in *Nodal* and *Lefty1* null embryos: (A) Volume rendering of Dvl2 in a representative 6.25 dpc wild-type littermate of the embryo in (C) showing membrane enrichment of Dvl2 in the Epi-VE and near absence in the ExE-VE. (A') Volume rendering of F-actin in the same embryo, showing localisation in sub-cortical rings in the Epi-VE and apical localisation in the ExE-VE. (B) Dvl2 localisation in a 5.5 dpc *Nodal* mutant showing dramatically reduced membrane localisation of Dvl2. (B') F-actin localisation in the same *Nodal* mutant showing absence of the apical actin shroud in the ExE-VE. (C) Volume rendering of Dvl2 localisation in a relatively mild 6.25 dpc *Nodal* mutant showing reduced membrane localisation of Dvl2 in the Epi-VE and abnormal persistence in the ExE-VE. (C') F-actin localisation in the same *Nodal* mutant showing the absence of an apical shroud in the ExE-VE. (D) Volume rendering of Dvl2 localisation in a 6.25 dpc *Lefty1* mutant showing ectopic membrane localisation of Dvl2 in the ExE-VE. (D') Volume rendering of F-actin localisation in the same *Lefty1* mutant showing attenuation of the apical actin shroud in the ExE-VE. (E) Volume rendering of Dvl2 localisation in the Epi-VE of a 5.5 dpc embryo cultured overnight in control medium. Dvl2 is membrane enriched as in normal uncultured embryos. (F) Membrane enrichment of Dvl2 is lost in embryo cultured overnight in the presence of the Nodal inhibitor SB431542. (G) Dvl2 localisation in an embryonic explant cultured overnight. Membrane enrichment of Dvl2 localisation is lost. (G') A high magnification image of the boxed region in (G). (H, H') Volume rendering and optical section of a 6.25 dpc *Lefty1* null mutant showing an AVE over-migration phenotype. (I, I') Volume rendering and optical section of a 6.25 dpc *Lefty1* null mutant showing an AVE arrest phenotype. Cell outlines in (H), (H'), and (I) are visualised by a Par-3 stain (grey) and nuclei in (I') with DAPI (grey). AVE cells are in green in all four panels. The lines in (H) and (H') indicate the border between the Epi-VE and ExE-VE. Scale bars  = 50 µm.

In opacity renderings, *Nodal* null mutants showed a dramatic failure to localise Dvl2 to the lateral membrane of Epi-VE cells and downregulate or exclude it from the membrane of ExE-VE cells (*n* = 5 out of 7) (Figure 6B). Even in slightly more mature mutant embryos that from their relatively intact morphology appeared to be less severely affected, Dvl2 was abnormally diffuse in the Epi-VE and persistent in the ExE-VE (Figure 6C). In the epiblast, Dvl2 was abnormally downregulated (Figure S4B and C). F-actin localisation was also abnormal in mutants, the apical shroud being essentially lost (Figure 6B' and C'). All *Nodal^lacZ/+^* heterozygotes examined had normal Dvl2 and F-actin localisation (*n* = 10).

These results suggest that Nodal activity is required for normal Dvl2 localisation. To further test this, we cultured 5.5 dpc embryos overnight in SB431542, a specific inhibitor of Nodal signalling [7],[41]. Control cultured embryos showed normal membrane enrichment of Dvl2 in the Epi-VE (*n* = 6). However, embryos cultured in the presence of inhibitor failed to enrich Dvl2 to the membrane (*n* = 7) (Figure 6E, F).

It has previously been shown that if the ExE and overlying VE are microsurgically removed from a 5.5 dpc embryo and the resulting embryonic explant cultured overnight, *Nodal* expression is lost in the epiblast and AVE cells fail to show migratory activity [6]. We looked at Dvl2 localisation in such explants (*n* = 6) and found that Dvl2 failed to become membrane enriched (Figure 6G, G').

Lefty1 is a secreted inhibitor of Nodal. In the absence of Lefty1, one would expect there to be increased Nodal signalling and increased or ectopic membrane enrichment of Dvl2. To test this hypothesis, we looked at Dvl2 in mutants for *Lefty1* [11]. Embryos were dissected at 6.25 dpc, stained for Dvl2, scanned, and opacity rendered. All the *Lefty1* homozygous null embryos examined (*n* = 4) had an abnormal persistence and enrichment of Dvl2 in the lateral cell membrane in the ExE-VE (compare Figure 6A and D). This defect was also seen in a few heterozygous mutants (2 out of 8). Dvl2 was expressed as normal in the epiblast of mutants but, in addition, was abnormally expressed in the ExE as well (Figure S4D). *Lefty1* mutants also showed an attenuation of the apical F-actin shroud (compare Figure 6A' and D').

The earliest phenotype reported for *Lefty1* null mutants is at 8.5 dpc [11]. To determine if the altered Dvl2 localisation in *Lefty1* null mutants might lead to an undetected AVE migration phenotype, we examined 6.25 dpc *Lefty1* null embryos carrying the *Hex-GFP* transgene and stained for Par-3 a marker of epithelial polarity that shows cell outlines. Of the seven such embryos examined, five had an AVE migration defect (*p* = 0.02, chi-square test, compared to four wild-type littermates). One of the five showed an arrest in AVE migration (Figure 6I, I'), while the remaining four showed an over-migration phenotype (Figure 6H, H„ and Movie S11). All showed the normal localisation pattern of Par-3. Among 13 heterozygotes examined, five had mild AVE migration defects (one showed a slight delay and four had slightly over-migrated). Among the total of eight embryos with an “over-migration” phenotype, six had been stained for F-actin. Four of these six showed a loss or attenuation of the F-actin shroud.

## Discussion

### Regional Differences in Cell Behaviour in the VE

The AVE and surrounding VE retain epithelial integrity during AVE migration. By using DIC optics to follow non-AVE cells of the VE, we show that AVE cells are not unique in their migratory movement and that surrounding cells of the Epi-VE also show a great deal of movement. We observe considerable cell intercalation in the Epi-VE (including among AVE and non-AVE cells). Migeotte et al. have recently reported cell intercalation among AVE cells [28]. Together, these suggest that AVE cells move through an intact VE epithelium by cell intercalation.

AVE cells stop migrating proximally upon reaching the border between the Epi-VE and ExE-VE and appear to become “squashed” against the ExE-VE and displaced laterally [29]. Cell tracking shows that Epi-VE cells ahead of the AVE show directional movement very similar to AVE cells, first moving proximally up to the ExE-VE and then getting squashed and displaced laterally. This suggests the stereotypic movement of AVE cells is part of a broader process of movement among all the cells of the Epi-VE.

AVE cells show protrusions predominantly in the direction in which they move [29]. These cellular protrusions have recently been shown to extend from the basal aspect of AVE cells [28]. It is possible that these protrusions sense a guidance cue like Dkk1 [9], which provides directional information to AVE cells, that then move through the intact VE epithelium by cell intercalation. Since non-Hex-GFP cells of the Epi-VE can only be tracked by DIC contrast of their apical surface (as opposed to Hex-GFP cells in which the entire cytoplasm is labelled), we cannot determine if they too show basal cellular projections. If the mode of movement of these cells is similar to that of AVE cells, one would predict that they also show directional cellular projections.

In contrast to the Epi-VE, cells in the ExE-VE show a marked absence of intercalation and are relatively static in shape and position. There are therefore two behaviourally distinct regions in the VE, consistent with differences in marker gene expression in these two regions that indicate they are differently patterned [7]. Like AVE cells, other Epi-VE cells “ahead” of the migrating AVE are also unable to move into the ExE-VE. This suggests that the inability of AVE cells to migrate into the ExE-VE is not an autonomous property of AVE cells caused, for example, by a change in their character during the course of migration leading them to stop at the ExE-VE. Rather, it appears to be a feature of the cellular context within which they move and a result of the inability of Epi-VE cells to intercalate with cells of the ExE-VE. In this view, surrounding VE cells influence the migration of AVE cells, both by providing a “permissive” environment where migration can take place (by allowing cell intercalation and changes in cell shape as in the Epi-VE) as well as by creating a non-permissive environment to halt proximal migration (by being less able to participate in intercalation and change shape as in the ExE-VE).

### Regional Differences in Molecular Localisation in the VE

Exploiting opacity rendering techniques has enabled us to detect regional differences in protein localisation in the VE that might otherwise have been missed. Differences in behaviour of cells in the VE are mirrored by differences in the localisation of the molecular motors F-actin and myosin IIA. It is possible that the different localisation of these molecules might cause the different behaviour of cells, with cortical actin rings in the Epi-VE facilitating the intercalation required for AVE migration and elevated actin levels localised to an apical shroud in the ExE-VE “locking” these cells in place with respect to each other and forming a barrier to AVE migration. Consistent with this, we see a diminution of the actin shroud in the majority of *Lefty1* mutants in which AVE cells “breach” the normal end-point to migration and move into the ExE-VE. Moreover, mutants for *Nap1*, a regulator of F-actin dynamics, show AVE migration defects [27] and null mutants for myosin IIA have severe VE defects by 6.5 dpc and die by 7.5 dpc [42]. Furthermore, reduction of myosin IIA leads to a reduction in F-actin stress fibres and increased migration in cultured human foreskin fibroblast cells [43]. However, to establish a direct causal role for these two molecules in modulating cell intercalation behaviour in the VE, one would need to directly and specifically disrupt their localisation in embryos to determine if AVE migration is perturbed. It would also be interesting to characterise myosin IIA mutants at 5.5 dpc, to determine if AVE migration or Epi-VE cell intercalation is altered.

Further highlighting differences between the Epi-VE and ExE-VE, we find that during AVE migration, Dvl2 is membrane localised specifically in the Epi-VE, suggestive of active PCP signalling in this region. The levels and localisation of Dvl2 shift in the ExE-VE through a continuum, from being membrane enriched prior to AVE migration to being progressively downregulated in both the plasma membrane and cytoplasm as the AVE migrates until it is almost completely absent in the ExE-VE of 6.25 dpc embryos. In contrast, Dvl2 remains unchanged in the Epi-VE, being membrane enriched at all stages. This view of Dvl2 localisation dynamics is built on data from a series of fixed stages. To probe in more detail how Dvl2 localisation changes and AVE migration relate to one another, it will be interesting to perform time-lapse microscopy of embryos expressing a fluorescent Dvl2 fusion protein.

Expression of a membrane tethered C-terminal fragment of the mouse Celsr-1 protein disrupts the membrane localisation of Dvl2 and Vangl2 in the Epi-VE, indicative of perturbed PCP signalling. Due to the widespread expression of the *ROSA26^Lyn-Celsr1^* transgene, the PCP disruption in the VE could be caused indirectly by effects in the epiblast or ExE. Such embryos show a range of AVE migration defects, consistent with a role for PCP signalling in AVE migration. The defect in Dvl2 and F-actin localisation in such embryos is subject to the dose of *ROSA26^Lyn-Celsr1^* (all homozygotes are affected, while only half the heterozygotes are affected) consistent with it being a specific effect of the *ROSA26^Lyn-Celsr1^* modification. The abnormal cytoplasmic aggregates of actin in these embryos are possibly the result of actin normally destined for the apical cortical domain instead accumulating in the cytoplasm. These embryos produce viable offspring despite defects in AVE migration, indicating that the embryo is able to accommodate a surprising amount of imprecision in AVE position during anterior pattering.

The phenotype of the *ROSA26^Lyn-Celsr1^* line is mild, even though this construct recapitulates the convergent extension defects caused by the zebrafish version when injected into zebrafish embryos. This difference in outcome in zebrafish and mouse could be due to a combination of reasons: the Lyn-Celsr1 construct is a mild dominant negative and injecting it in zebrafish allows one to express it at higher levels than can be achieved from the *ROSA26* locus in mice, which is not expressed at particularly high levels; differences in how PCP signalling is set up in zebrafish and mouse might make the former more susceptible to disruption. In support of the latter possibility is the reported functional redundancy in PCP signalling at later stages of mouse embryogenesis for the key PCP molecules Dvl-1, Dvl2, and Dvl-3 [44]. Since AVE migration is vital for the further development of the embryo, it would not be surprising if there is redundancy to the mechanisms regulating it.

Mutants of PCP pathway genes generally have phenotypes at relatively late stages in development, for example in the organ of Corti, neural tube, or developing heart. To date, no pre-gastrulation defects have been reported in such mutants, contrary to what one might expect if PCP signalling was involved in AVE migration. However, given that *ROSA26^Lyn-Celsr1^* and *Lefty1* embryos do not show any obvious patterning defects at 5.5 dpc despite mislocalisation of Dvl2 and perturbed AVE migration, it is possible that a similarly subtle non-lethal early phenotype has been missed in these PCP mutants. For example, Dvl1−/−, Dvl2−/−, and Dvl3−/− triple mutants have impaired gastrulation [45], consistent with a possible AVE defect.

### Nodal Signalling Is Required for Membrane Localisation of Dvl2

Consistent with experiments in the Xenopus animal cap that show that Activin can lead to membrane localisation of Dishevelled, membrane enrichment of Dvl2 is lost in the VE of *Nodal* mutants. This result is unlikely to be simply due to a general breakdown of epithelial organisation of the VE because the VE of *Nodal* null mutants continues to express E-cadherin as normal [7], indicating it retains epithelial characteristics. Furthermore, similar results were observed in embryos cultured in the Nodal inhibitor SB431542 and in explants where the ExE had been removed, which downregulate *Nodal* in the epiblast. In both N*odal* mutants and SB431542 treated embryos, the entire VE appears uniform with respect to Dvl2 localisation, consistent with a role for Nodal in setting up regional identity in the VE suggested by Mesnard et al. [7].

As one might predict, if *Nodal* had a specific effect on the membrane localisation of Dvl2, we see ectopic membrane localisation of Dvl2 in mutants of the *Nodal* inhibitor *Lefty1.* Moreover, *Lefty1* mutants show otherwise normal VE epithelial morphology, including normal expression of Par-3, a marker of epithelial polarity, suggesting that the VE is not broadly disrupted and consistent with the effect of Nodal and Lefty1 on Dvl2 localisation being a specific one. In both mutants, we see effects in the ExE-VE, though *Nodal* is expressed in the epiblast, and *Lefty1* is expressed in the AVE. Both are secreted molecules and might diffuse and act directly on the ExE-VE (in addition to on the Epi-VE). While our results point to a specific interaction between the Nodal and PCP signalling pathways, it remains unclear whether this interaction is direct (as in Nodal and PCP signalling pathways having components that interact, leading to cross-talk) or indirect (as in requiring the activation/repression of a Nodal target gene that then affects PCP signalling).

**Figure 7:**
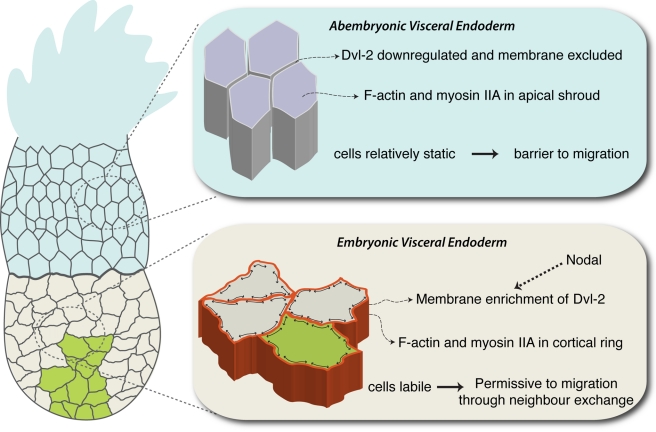
Model of summarising differences in cell intercalation in the VE. Migrating AVE cells are shown in green. The lower blow-up shows Epi-VE cells in which Dvl2, F-actin and Myosin IIA are localised in lateral cortical rings. These cells are labile (depicted by the black double-headed arrows within the cell boundary), which facilitates AVE migration by cell intercalation. In contrast, in the ExE-VE, Dvl2 is excluded from the membrane and F-actin is localised to an apical shroud. These cells are static in shape and do not intercalate, creating a barrier to migration. Nodal signalling directly or indirectly influences the membrane localisation of Dvl2.

In conclusion, we suggest a model where there are two behaviourally distinct regions in the VE, one with exuberant cell movements in which AVE cells migrate by cell intercalation and a static region in which migration is not possible. These two regions are characterised by differences in localisation of Dvl2, F-actin, and myosin IIA, regulated by interplay between Nodal and PCP signalling (Figure 7). The interface between these two regions of different behaviour creates an effective barrier to AVE migration that can be disrupted by perturbing Dvl2 localisation. The dynamic pattern of Dvl2 localisation is directly or indirectly dependent on Nodal signalling, providing a mechanism whereby this key patterning molecule might also modulate cell behaviour in epithelia.

## Materials and Methods

### Mouse Strains, Husbandry, and Embryo Collection

Genetically modified mice were maintained on a mixed C57Bl/6 CBA/J background. The *Hex-GFP* line was bred into the various mutant backgrounds to enable the AVE to be followed. Wild-type embryos carrying the *Hex-GFP* transgene were obtained by crossing homozygous *Hex-GFP* studs with CD1 females (Charles River). All mice were maintained on a 12 h light, 12 h dark cycle. Noon on the day of finding a vaginal plug was designated 0.5 dpc. Embryos of the appropriate stage were dissected in M2 medium (Sigma) with fine forceps and tungsten needles. For embryo explant experiments, the ExE and overlying ExE-VE were removed using tungsten needles.

### Embryo Culture and Time-Lapse Microscopy

Culture media consisted of 50% home-made heat-inactivated mouse serum and 50% CMRL (Invitrogen) supplemented with L-glutamine, equilibrated at 37°C and 5% CO_2_ for at least 2 h prior to use. For Nodal inhibition experiments, SB431542 (Sigma 54317) was dissolved in DMSO to make a 10 mM stock solution and used as a 1∶1000 dilution in culture medium to give a 10 µM final working concentration. Control embryos for the inhibitor experiment were cultured in culture medium with 1∶1000 DMSO (the carrier for SB431542).

For time-lapse experiments, embryos were transferred into the pre-equilibrated media in Lab-TekII Coverglass bottomed eight well rectangular chambers (Nalge Nunc International) and imaged for up to 8 h on an inverted Zeiss 710 confocal microscope equipped with an environmental chamber to maintain conditions of 37°C and 5% CO2. Embryos were imaged with a water immersion 40X/1.2 NA objective every 15 min. At every time-point, a Z-stack of 5 focal planes separated by 10.78 µm was captured. EGFP marking AVE cells was excited at 488 nm and DIC images were acquired with the confocal's transmitted light PMT.

### Cell Tracking and Quantification

VE cell outlines were manually traced using Volocity software (Improvosion UK), by examining multiple focal planes for each time-point. Cells were outlined only if their apical margins could be unambiguously discerned and individual cells were followed on average for 12 consecutive time-points.

Cell tracks were generated by Volocity on the basis of the centroids of outlined cells.

Neighbour exchange was defined as the gain or loss of contact between two cells at consecutive time-points, with each loss or gain scored as 1. Exchange events were scored separately for the Epi-VE and ExE-VE (46 and 44 cells, respectively, from 4 embryos) at each time interval and then normalised by divided by the number of cells in each region for that time interval. Normalised exchange events were then averaged across all the time-intervals considered.

The average cell movement in the Epi-VE and ExE-VE (46 and 44 cells, respectively, from 4 embryos) was calculated as follows. The distance moved by a cell in any one time-interval was calculated as the difference in position of its centroid at two consecutive time-points. The distance moved by cells in the Epi-VE and ExE-VE was then averaged for all the time intervals of the time-lapse data.

Shape factor is a numeric value between 0 and 1, and is a measure of the distortedness of a cell's shape. Regular shapes like hexagons and pentagons generally score higher than flattened or irregular shapes. The shape factor of cells in the Epi-VE and ExE-VE (46 and 44 cells, respectively, from 4 embryos) was calculated using Volocity and averaged for all the time intervals of the time-lapse data.

### Immunohistochemistry

The specificity of the Dvl2 antibody was verified using a blocking peptide (Biomol DP9427). Secondary only controls were done to verify the specificity of secondary antibodies.

Embryos were fixed in 4% PFA in PBS at 4°C for 30 min (except for Dvl2 stains, see below); washed at room temperature thrice for 5 min each in 0.1% Triton-X100 in PBS (PBT); incubated in 0.25% Triton-X100 in PBS for 15 min; washed thrice in PBT; blocked with 2.5% donkey serum, 2.5% goat serum, and 3% Bovine Serum Albumin (BSA) in PBT for 1 h; incubated overnight at 4°C in primary antibodies diluted in 100 µl PBT; washed five times in PBT for 5 min each, with a final additional wash for 20 min; incubated at room temperature in the appropriate secondary diluted in 100 µl PBT for 2 h or overnight; washed in PBT five times for 5 min and once for 15 min; and finally mounted with Vectashield mounting media containing DAPI (Vector Labs H-1200).

If staining with Phalloidin for F-actin, embryos were fixed and washed as described above, incubated in Phalloidin diluted in 100 µl PBT for 1 h at room temperature, and then washed as described above for washing after the secondary. Phalloidin was also used to stain antibody stained embryos, by incubating embryos in 100 µl diluted Phalloidin for 1 h at room temperature immediately after the first wash after incubating in secondary. The embryos were then washed as described above.

For Dishevelled-2 staining, embryos were fixed in 4% PFA in PBS containing 130 mM KCl, 25 mM HEPES, 3 mM MgCl_2_, 0.15% glutaraldehyde, and 0.06% Triton-X100. After the blocking step Tween-20 was used instead of Triton-X100 in all washes.

### Antibodies and Phalloidin

Primary antibodies used were: Rabbit anti-ZO-1 (Zymed laboratories 61-7300) 1∶100, Rabbit anti-Myosin IIA (Sigma M8064) 1∶100, Rat Anti-Uvomorulin/E-cadherin (Sigma U3254) 1∶200, Rabbit anti-Dishevelled-2 (Biomol 4270) 1∶200, Sheep anti-Vangl2 (R&D Systems AF4815) 1∶100, and Rabbit anti-Par-3 (Millipore 07-330) 1∶100. The secondary antibodies used were: Alexa Fluor 555 donkey anti-rabbit IgG (Invitrogen A-31572), Northern Lights 637 anti-sheep IgG (R&D Systems NL011), and Alexa Fluor 633 goat anti-rat IgG (Invitrogen A-21094). For F-actin stains, either TRITC-Phalloidin (Sigma 77418) at a final concentration of 1 mg/ml or Atto 647N-Phalloidin (Sigma 65906) at a final concentration of 1 nM were used.

### Confocal Microscopy and Volume Rendering

Fixed samples were imaged on Zeiss LSM 510META and Zeiss LSM 710 confocal microscopes using 20X/0.75NA or 40X/1.2NA lenses as appropriate. DAPI was excited at 405 nm, EGFP at 488 nm, Alexa Fluor 555 at 543 nm, Alexa Fluor at 633 nm, and Atto647N-Phalloidin at 633 nm. Z-stacks of entire embryos were acquired at a 0.8 um interval using non-saturating scan parameters. Z-stacks of embryos were opacity rendered as 3D volumes using Volocity Software (Improvision, UK). Extended focus projections were maximum intensity projections. Figures were prepared with Adobe CS2 Photoshop and Illustrator (Adobe Inc).

### Generation of ROSA26^Lyn-Celsr1^ Mice

The targeting construct was made by inserting a *Lyn-Celsr1-EGFP* fusion gene into pBigT and pROSA26PA (details of molecular cloning available on request) [46]. This construct was used to modify the ROSA26 locus of ES cells by homologous recombination. The construct contained a LoxP flanked transcriptional stop cassette, such that expression of *Lyn-Celsr1-EGFP* was conditional on Cre expression. ES cells were subject to positive selection for neomycin resistance and negative selection against DTA. ES cells were screened using the Southern Blot and positive clones used to produce germline chimeras by blastocyst injection. Mice inheriting the transgene through the germline were crossed to a βactin-Cre line [47] to excise the floxed transcriptional stop cassette in all tissues. Such mice were bred to segregate out the β-actin transgene and establish the *ROSA26^Lyn-Celsr1^* line that expresses *Lyn-Celsr1-EGFP* ubiquitously and constitutively.

### Embryo Genotyping

Antibody stained confocal imaged embryos were recovered from slides, washed in syringe filtered PBT thrice for 5 min, washed in lysis buffer (50 mM Tris HCl pH 8–8.5, 1 mM EDTA, 0.5% Tween-20) for 5 min, transferred into PCR strips containing lysis buffer (16 µl for 5.5 dpc embryos) and Proteinase K (1 µl 20 mg/ml PK per 25 µl of embryo lysis buffer), lysed at 55°C for 1 h, and the ProteinaseK inactivated by incubating at 95°C for 10 min. PCR genotyping was performed using 3 µl of lysed embryo as template, the appropriate primers, and Illustra puReTaq Ready-To-Go PCR Beads (GE Healthcare Catalogue No. 27-9557-01 (0.2 ml tubes/plate 96)).

### PCR Primers and Conditions

Primers for *ROSA26^Lyn-Celsr1-GFP^*: R1 – 5′AAAGTCGCTCTGAGTTGTTAT3′; R2 – 5′GCGAAGAGTTTGTCCTCAACC3′; R3 – 5′GGAGCGGGAGAAATGGATATG3′. Bands expected: 250 bp mutant (R1+R2) and 500 bp wild-type (R1+R3).

Primers for *Nodal^lacZ^*: LacZ-5′ – 5′CCGCGCTGTACTGGAGGCTGAAG3′; LacZ-3′ – 5′ATACTGCACCGGGCGGGAAGGAT3′; A – 5′ATGTGGACGTGACCGGACAGAACT3′; B – 5′CTGGATGTAGGCATGGTTGGTAGGAT3′. Bands expected: 750 bp mutant and 700 bp wild-type.

Primers for Lefty: 148 – 5′CAGGCATCCAGCAGAGAACG3′; 149 – 5′AGGGCTTCCCTGAGGCTAAC3′; 150 – 5′ACCCAGCACTCCACTGGATA3′. Bands expected: 700 bp mutant and 500 bp wild-type.

## Supporting Information

Figure S1 Dvl2 localisation in later stage embryos. (A, B) Volume rendering of F-actin and Dvl2 localisation in a representative 6.25 dpc embryo. Hex-GFP labelled AVE cells are shown in green. F-actin is present in cortical rings in the Epi-VE and in an apical shroud in the ExE-VE. Dvl2 is membrane enriched in the Epi-VE and only present at very low levels in the ExE-VE. (C) Merged rendering of F-actin (grey), Dvl2 (magenta), and Hex-GFP labelled AVE cells (green) at 6.25 dpc. (D, E, F) F-actin and Dvl2 localisation in a representative 7.5 dpc embryo with Hex-GFP labelled AVE and anterior definitive endoderm cells in green. Dvl2 is membrane enriched in the Epi-VE and almost absent in the ExE-VE. F-actin is predominantly apical in the ExE-VE, and predominantly in cortical rings in the Epi-VE. Scale bars  = 100 µm.
(TIF)

Figure S2 Generation of the *ROSA26^Lyn-Celsr1^* mouse line. (A) Diagram illustrating the structure of the Celsr1 protein, and that a C-terminal truncated version of the protein disrupts PCP signalling. (B) The fusion gene contains a myristoylation signal from the Lyn kinase (to target the protein to the inner leaflet of the plasma membrane), the C-terminal domain of mouse Celsr-1 (to disrupt PCP signalling), and EGFP to monitor localisation of the fusion protein. Expression of the truncated mouse Celsr1 in zebrafish embryos results in a convergent extension defect, indicative of impaired PCP signalling. The phenotype was classed as mild or severe according to the extent of the defects observed. RNA in situ hybridisation on tail-bud stage embryos marking the anterior edge of the neural plate (distal-less3), the prechordal plate (cathepsin-L), and the notochord (no tail). A schematic of the tailbud stage embryo, on the left, shows the location of these structures; Pre, Prechordal plate; NP, Neural Plate; Ntc, Notochord. Anterior is to the top and posterior is to the bottom. Marker analysis revealed that, compared to control uninjected embryos, those injected with Lyn-Celsr1-GFP have a wider neural plate, a posteriorised prechordal plate, and a wider notochord. Embryos expressing a control membrane tethered GFP did not demonstrate any developmental abnormalities. (C) Schematic outlining the strategy used to target the ROSA26 locus. (D) Southern blot analysis of genomic DNA from a correctly targeted clone digested with Eco RV, StuI, and BamHI, probed with a 5′ external probe. All three digests produced correct bands for the wild-type allele (upper bands) and targeted allele (lower bands). (E) Expression of *ROSA26^Lyn-Celsr1^* in a 5.5 dpc embryo. Confocal sections show the membrane localised Lyn-Celsr1-GFP fusion protein expressed throughout the VE, epiblast, and ExE. (F) Cer1-1 expression marking the AVE in a *ROSA26^Lyn-Celsr1^* embryo, showing cells abnormally extending onto the ExE. (G) Optical section of a *ROSA26^Lyn-Celsr1^* embryo showing Eomesodermin expression (magenta) and DAPI stained nuclei (grey). Eomesodermin is localised normally in the posterior epiblast, ExE, and VE.
(TIF)

Figure S3 Vangl2 localisation is disrupted in *ROSA26^Lyn-Celsr1^* embryos. Volume rendering of the core PCP protein Vangl2 in 6.25 dpc wild-type (A) and *ROSA26^Lyn-Celsr1^* (B) embryos. In wild-type embryos Vangl2 is membrane enriched in the Epi-VE and somewhat more diffuse in the ExE-VE. In *ROSA26^Lyn-Celsr1^* embryos, Vangl2 is downregulated and not membrane enriched in the Epi-VE. (A', B') High magnification views of the boxed-in regions in (A) and (B), respectively. Scale bar  = 50 µm.
(TIF)

Figure S4 Optical sections of Dvl2 expression in *Nodal* and *Lefty1* mutants. (A) 6.25 dpc wild-type embryo. (B) 5.5 dpc *Nodal^lacZ/lacZ^* mutant. (C) 6.25 dpc *Nodal^lacZ/lacZ^* mutant littermate of the embryo in (A). (D) 6.25 dpc *Lefty1* mutant. In the *Nodal* mutants, Dvl2 expression is slightly reduced in the epiblast, particularly at 6.25 dpc. In *Lefty1* mutants, Dvl2 is abnormally upregulated in the ExE. Scale bars  = 50 µm.
(TIF)

Movie S1 Comparison of raw image stack versus volume rendering of 3D conofocal image data. ZO-1 (tight junctions) in magenta and nuclei (DAPI stained) in grey. Use keyboard arrow keys to navigate frame by frame through the animation.
(MOV)

Movie S2 ZO-1 and E-cadherin in the VE. Volume rendering of a WT embryo, with ZO-1 (tight junctions) in magenta, E-cadherin (adherens junctions) in yellow, AVE cells (marked by Hex-GFP expression) in green, and nuclei (DAPI stained) in grey. Use keyboard arrow keys to navigate frame by frame through the animation.
(MOV)

Movie S3 Time-lapse of neighbour exchange during AVE migration. Time-lapse sequence of neighbour exchange in the embryonic VE during AVE migration. Images were taken every 15 min. The embryo is orientated with anterior towards the viewer. AVE cells are marked by *Hex-GFP* expression (green). Selected cells have been outlined to help follow them. Neighbour exchange is observed only in the Epi-VE. Epi-VE cells also change shape continuously, in contrast to relatively static ExE-VE cells. Use the keyboard arrow keys to navigate frame by frame.
(MOV)

Movie S4 Tracks of VE cells in different regions of the embryo. Time-lapse sequence of an embryo showing differences in behaviour between Epi-VE and ExE-VE cells. Images were taken every 15 min. The embryo is orientated with anterior towards the viewer. AVE cells are marked by Hex-GFP expression (green) in the left-hand side panel. In the panel to the right, the GFP channel has been removed and cells outlined to help follow them. AVE cells and their projections are in green, non-AVE Epi-VE cells are magenta, and ExE-VE cells are blue. The basal projections of AVE cells can be seen “overlapping” with the magenta Epi-VE cells ahead of them. The last frame shows the tracks of the marked cells. Use the keyboard arrow keys to navigate frame by frame.
(MOV)

Movie S5 Time-lapse of cell deformation during AVE migration. Time-lapse sequence of cell changes in the Epi-VE late during AVE migration. Images were taken every 15 min. The embryo is orientated with anterior towards the viewer. AVE cells are marked by *Hex-GFP* expression (green). Selected cells have been outlined to help follow them. Neighbour exchange is observed only in the Epi-VE. Epi-VE cells undergo dramatic changes in shape, in contrast to the relatively static ExE-VE cells. Use the keyboard arrow keys to navigate frame by frame.
(MOV)

Movie S6 F-actin localisation in a wild-type embryo. Volume rendering of a wild-type embryo, with F-actin in yellow, AVE cells in green (marked by *Hex-GFP* expression), and nuclei in grey (DAPI stained). F-actin is localised to cortical rings in cells of the Epi-VE, but to an apical shroud in the ExE-VE. Use keyboard arrow keys to navigate frame by frame through the animation.
(MOV)

Movie S7 F-actin in a *ROSA26^Lyn-Celsr1^* embryo. Volume rendering of F-actin in a *ROSA26^Lyn-Celsr1^* transgenic embryo. The transparency of F-actin is progressively increased to show features otherwise obscured by the surface. This reveals cytoplasmic aggregates of F-actin, marked by arrow-heads. AVE cells are labelled green by the expression of Hex-GFP. They abnormally cross onto the ExE. Use keyboard arrow keys to navigate frame by frame through the animation.
(MOV)

Movie S8 Abnormal AVE migration in a *ROSA26^Lyn-Celsr1^* embryo. Volume rendering of a *ROSA26^Lyn-Celsr1^* transgenic embryo, with ZO-1 in magenta (tight junctions, showing cell outlines), AVE cells in green (marked by Hex-GFP expression), and nuclei in grey (labelled with DAPI). AVE cells have migrated abnormally, onto the ExE. Use keyboard arrow keys to navigate frame by frame through the animation.
(MOV)

Movie S9 Abnormal AVE migration in a *ROSA26^Lyn-Celsr1^* embryo. Volume rendering of *ROSA26^Lyn-Celsr1^* transgenic embryo, with ZO-1 in magenta (tight junctions, showing cell outlines) and AVE cells in green (marked by Hex-GFP expression). AVE cells have migrated abnormally widely throughout the Epi-VE and also onto the ExE. Use keyboard arrow keys to navigate frame by frame through the animation.
(MOV)

Movie S10 Abnormal AVE migration in a *ROSA26^Lyn-Celsr1^* embryo. Volume rendering of a *ROSA26^Lyn-Celsr1^* transgenic embryo, with ZO-1 in magenta (tight junctions, showing cell outlines) and AVE cells in green (marked by Hex-GFP expression). AVE cells have formed an abnormal swirl and have migrated with a strong bias towards the “left” of the embryo. Use keyboard arrow keys to navigate frame by frame through the animation.
(MOV)

Movie S11 Abnormal AVE migration in a *Lefty1* null embryo. Volume rendering of a *Lefty1* null embryo, with AVE cells in green, nuclei in grey, Par-3 in magenta, and F-actin in yellow. Par-3 and F-actin are used to show cell outlines. The boundary between the epiblast and ExE is marked by two magenta lines at the sides of the embryo. AVE cells have migrated abnormally onto the ExE. Use keyboard arrow keys to navigate frame by frame through the animation.
(MOV)

## Abbreviations

dpc: days *post coitum*
Dvl2: Dishevelled-2
Epi-VE: VE overlying epiblast
ExE: extraembryonic ectoderm
ExE-VE: VE overlying ExE
PCP: Planar Cell Polarity
VE: visceral endoderm
