# Supplementary figures and images for: Nodal Dependent Differential Localisation of Dishevelled-2 Demarcates Regions of Differing Cell Behaviour in the Visceral Endoderm
Source: PLoS Biol. 2011 Feb 22;9(2):e1001019. doi: 10.1371/journal.pbio.1001019 (PMC3042994; doi:10.1371/journal.pbio.1001019)

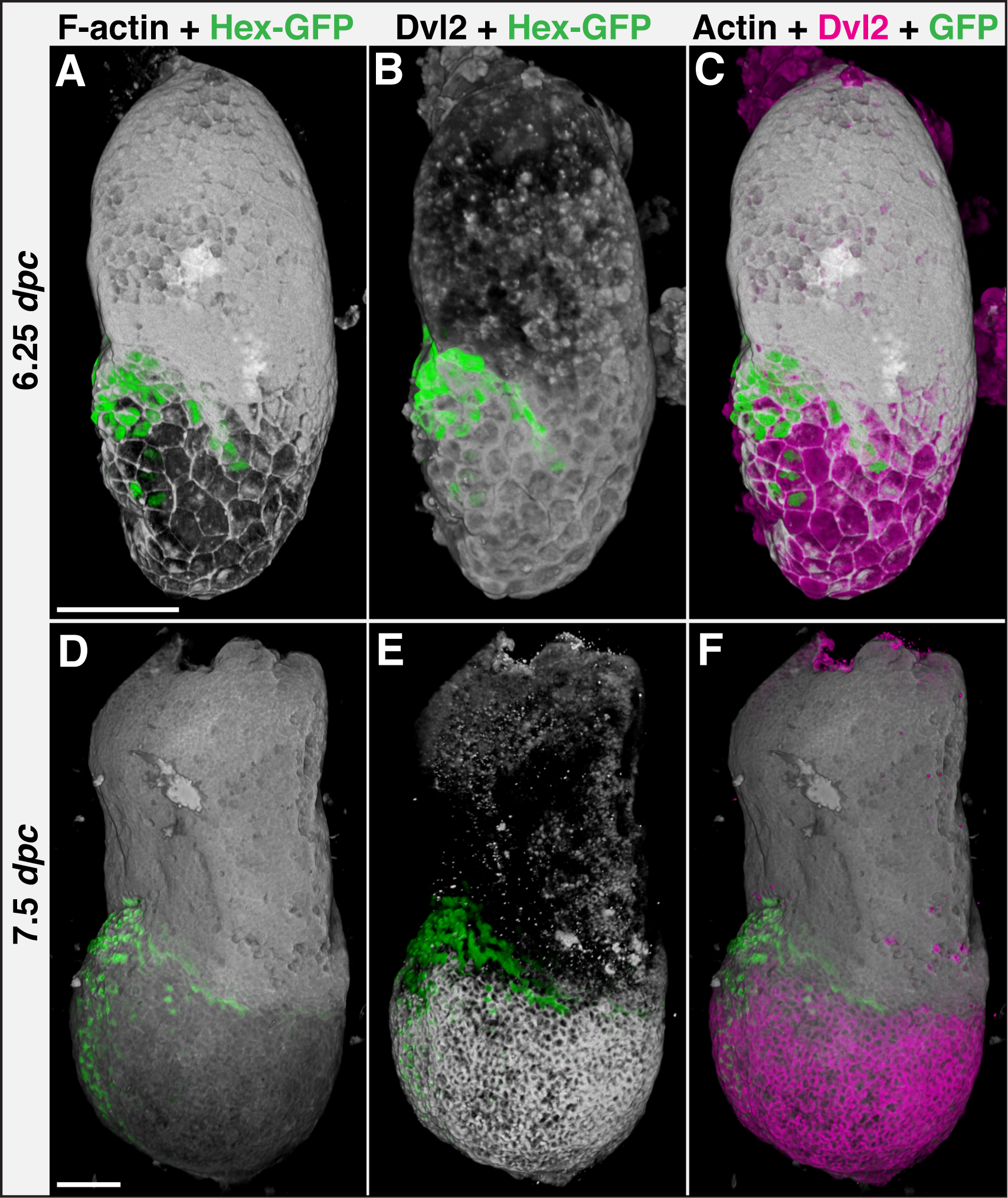

Supplement: Figure S1 — Dvl2 localisation in later stage embryos. (A, B) Volume rendering of F-actin and Dvl2 localisation in a representative 6.25 dpc embryo. Hex-GFP labelled AVE cells are shown in green. F-actin is present in cortical rings in the Epi-VE and in an apical shroud in the ExE-VE. Dvl2 is membrane enriched in the Epi-VE and only present at very low levels in the ExE-VE. (C) Merged rendering of F-actin (grey), Dvl2 (magenta), and Hex-GFP labelled AVE cells (green) at 6.25 dpc. (D, E, F) F-actin and Dvl2 localisation in a representative 7.5 dpc embryo with Hex-GFP labelled AVE and anterior definitive endoderm cells in green. Dvl2 is membrane enriched in the Epi-VE and almost absent in the ExE-VE. F-actin is predominantly apical in the ExE-VE, and predominantly in cortical rings in the Epi-VE. Scale bars = 100 µm. (TIF) [file pbio.1001019.s001.tif]

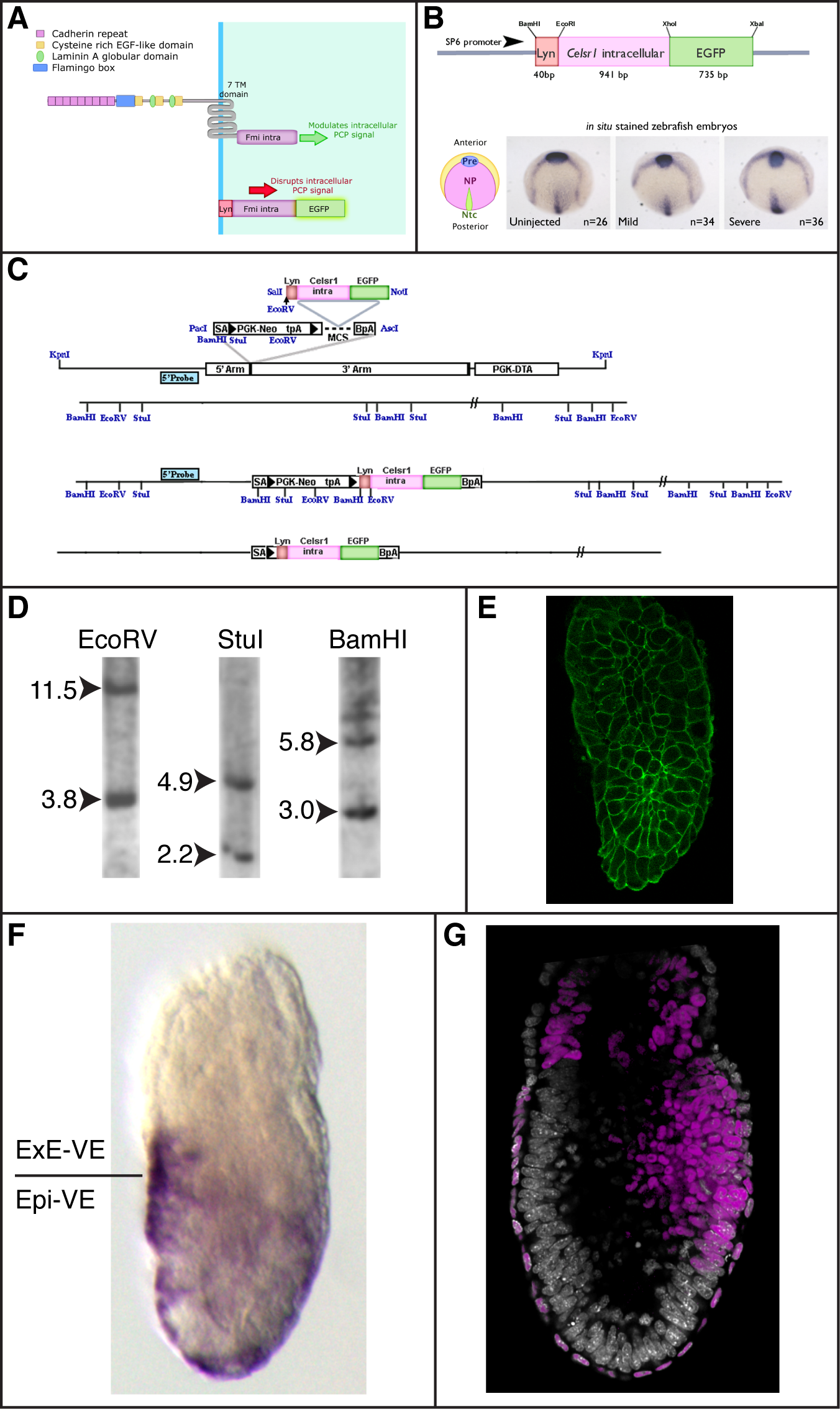

Supplement: Figure S2 — Generation of the ROSA26Lyn-Celsr1 mouse line. (A) Diagram illustrating the structure of the Celsr1 protein, and that a C-terminal truncated version of the protein disrupts PCP signalling. (B) The fusion gene contains a myristoylation signal from the Lyn kinase (to target the protein to the inner leaflet of the plasma membrane), the C-terminal domain of mouse Celsr-1 (to disrupt PCP signalling), and EGFP to monitor localisation of the fusion protein. Expression of the truncated mouse Celsr1 in zebrafish embryos results in a convergent extension defect, indicative of impaired PCP signalling. The phenotype was classed as mild or severe according to the extent of the defects observed. RNA in situ hybridisation on tail-bud stage embryos marking the anterior edge of the neural plate (distal-less3), the prechordal plate (cathepsin-L), and the notochord (no tail). A schematic of the tailbud stage embryo, on the left, shows the location of these structures; Pre, Prechordal plate; NP, Neural Plate; Ntc, Notochord. Anterior is to the top and posterior is to the bottom. Marker analysis revealed that, compared to control uninjected embryos, those injected with Lyn-Celsr1-GFP have a wider neural plate, a posteriorised prechordal plate, and a wider notochord. Embryos expressing a control membrane tethered GFP did not demonstrate any developmental abnormalities. (C) Schematic outlining the strategy used to target the ROSA26 locus. (D) Southern blot analysis of genomic DNA from a correctly targeted clone digested with Eco RV, StuI, and BamHI, probed with a 5′ external probe. All three digests produced correct bands for the wild-type allele (upper bands) and targeted allele (lower bands). (E) Expression of ROSA26Lyn-Celsr1 in a 5.5 dpc embryo. Confocal sections show the membrane localised Lyn-Celsr1-GFP fusion protein expressed throughout the VE, epiblast, and ExE. (F) Cer1-1 expression marking the AVE in a ROSA26Lyn-Celsr1 embryo, showing cells abnormally extending onto the ExE. (G [file pbio.1001019.s002.tif]

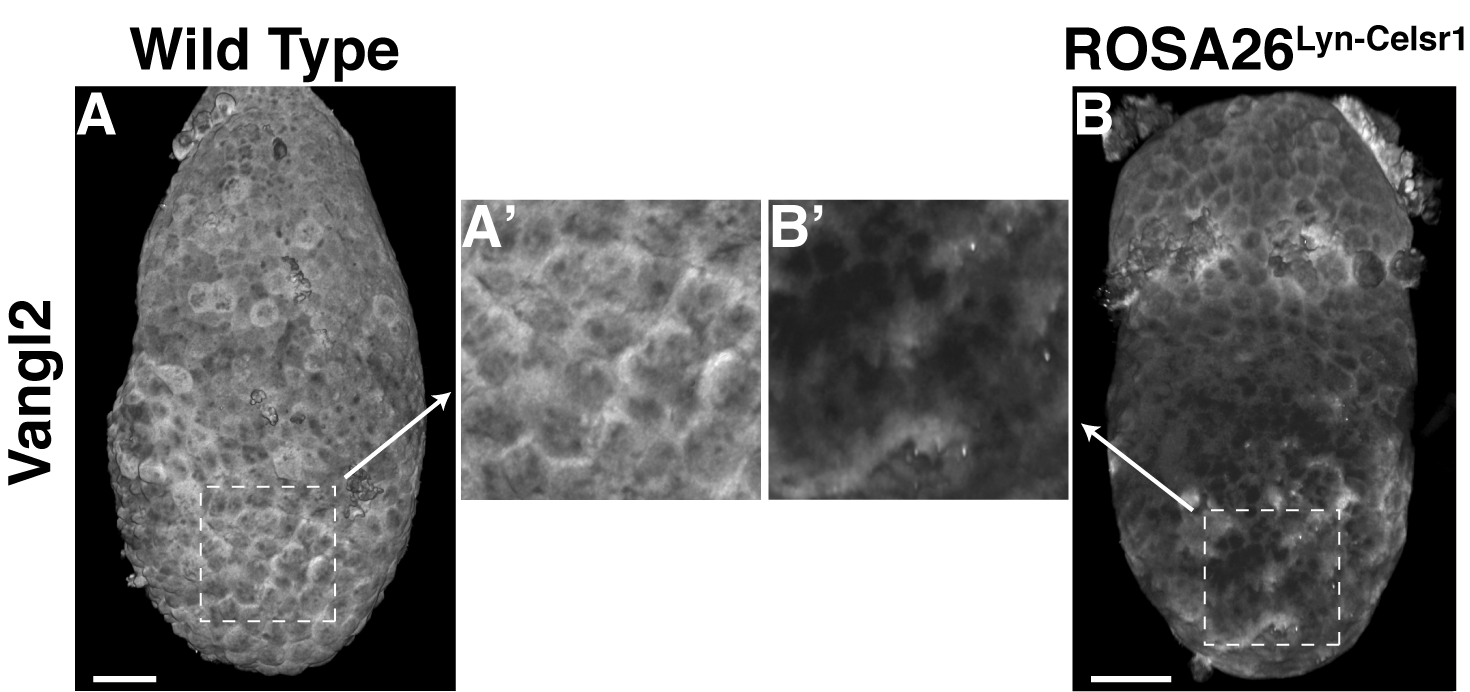

Supplement: Figure S3 — Vangl2 localisation is disrupted in ROSA26Lyn-Celsr1 embryos. Volume rendering of the core PCP protein Vangl2 in 6.25 dpc wild-type (A) and ROSA26Lyn-Celsr1 (B) embryos. In wild-type embryos Vangl2 is membrane enriched in the Epi-VE and somewhat more diffuse in the ExE-VE. In ROSA26Lyn-Celsr1 embryos, Vangl2 is downregulated and not membrane enriched in the Epi-VE. (A', B') High magnification views of the boxed-in regions in (A) and (B), respectively. Scale bar = 50 µm. (TIF) [file pbio.1001019.s003.tif]

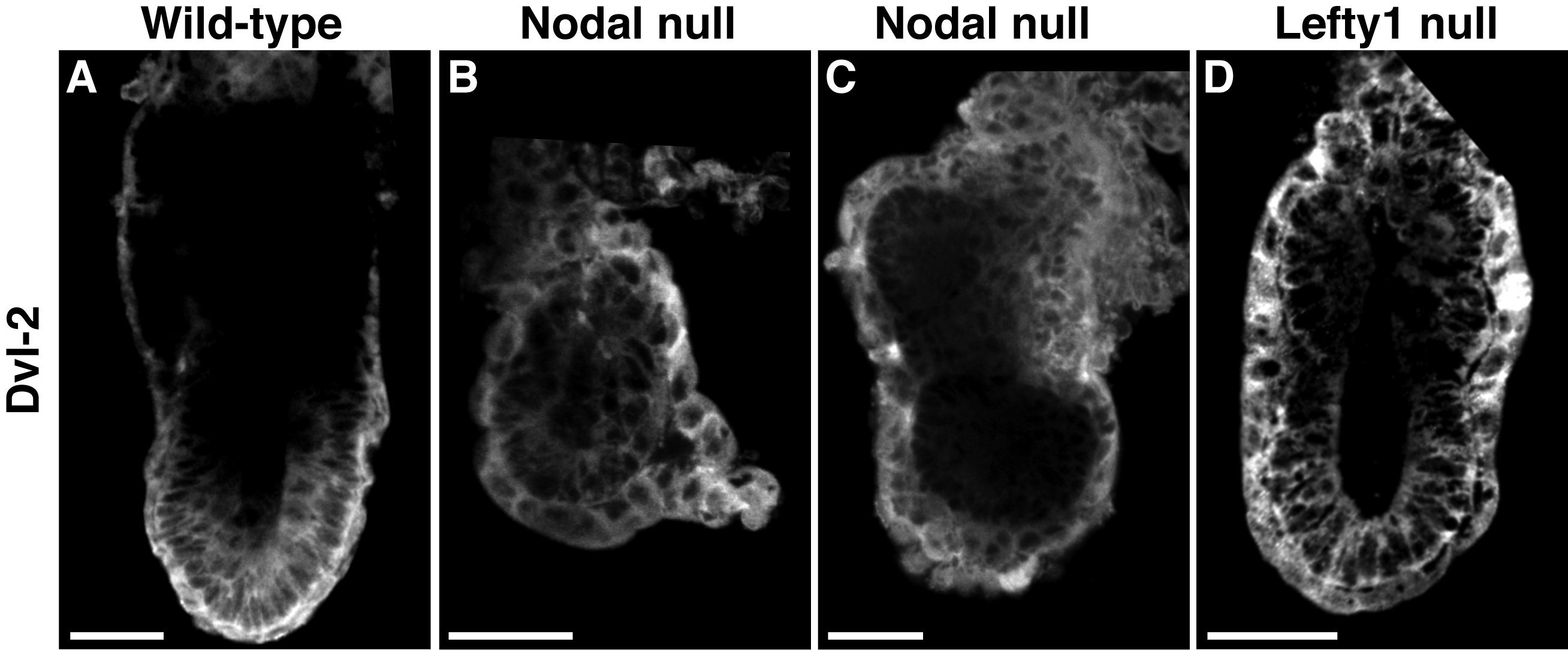

Supplement: Figure S4 — Optical sections of Dvl2 expression in Nodal and Lefty1 mutants. (A) 6.25 dpc wild-type embryo. (B) 5.5 dpc NodallacZ/lacZ mutant. (C) 6.25 dpc NodallacZ/lacZ mutant littermate of the embryo in (A). (D) 6.25 dpc Lefty1 mutant. In the Nodal mutants, Dvl2 expression is slightly reduced in the epiblast, particularly at 6.25 dpc. In Lefty1 mutants, Dvl2 is abnormally upregulated in the ExE. Scale bars = 50 µm. (TIF) [file pbio.1001019.s004.tif]
